# Quantitative analysis of changes in actin microfilament contribution to cell plate development in plant cytokinesis

**DOI:** 10.1186/1471-2229-8-80

**Published:** 2008-07-17

**Authors:** Takumi Higaki, Natsumaro Kutsuna, Toshio Sano, Seiichiro Hasezawa

**Affiliations:** 1 Department of Integrated Biosciences, Graduate School of Frontier Sciences, The University of Tokyo, Kashiwanoha Kashiwa, Chiba 277-8562, Japan; 2 Institute for Bioinformatics Research and Development (BIRD), Japan Science and Technology Agency (JST), Chiyoda-ku, Tokyo 102-8666, Japan

## Abstract

**Background:**

Plant cells divide by the formation of new cross walls, known as cell plates, from the center to periphery of each dividing cell. Formation of the cell plate occurs in the phragmoplast, a complex structure composed of membranes, microtubules (MTs) and actin microfilaments (MFs). Disruption of phragmoplast MTs was previously found to completely inhibit cell plate formation and expansion, indicative of their crucial role in the transport of cell plate membranes and materials. In contrast, disruption of MFs only delays cell plate expansion but does not completely inhibit cell plate formation. Despite such findings, the significance and molecular mechanisms of MTs and MFs remain largely unknown.

**Results:**

Time-sequential changes in MF-distribution were monitored by live imaging of tobacco BY-2 cells stably expressing the GFP-actin binding domain 2 (GFP-ABD2) fusion protein, which vitally co-stained with the endocytic tracer, FM4-64, that labels the cell plate. During cytokinesis, MFs accumulated near the newly-separated daughter nuclei towards the emerging cell plate, and subsequently approached the expanding cell plate edges. Treatment with an actin polymerization inhibitor caused a decrease in the cell plate expansion rate, which was quantified using time-lapse imaging and regression analysis. Our results demonstrated time-sequential changes in the contribution of MFs to cell plate expansion; MF-disruption caused about a 10% decrease in the cell plate expansion rate at the early phase of cytokinesis, but about 25% at the late phase. MF-disruption also caused malformation of the emerging cell plate at the early phase, indicative of MF involvement in early cell plate formation and expansion. The dynamic movement of endosomes around the cell plate was also inhibited by treatment with an actin polymerization inhibitor and a myosin ATPase inhibitor, respectively. Furthermore, time-lapse imaging of the endoplasmic reticulum (ER) revealed that MFs were involved in ER accumulation in the phragmoplast at the late phase.

**Conclusion:**

By expression of GFP-ABD2 and vital staining with FM4-64, the dynamics of MFs and the cell plate could be followed throughout plant cytokinesis in living cells. Pharmacological treatment and live imaging analysis also allowed us to quantify MF contribution to cell plate expansion during cytokinesis. Our results suggest that MFs play significant roles in cell plate formation and expansion via regulation of endomembrane dynamics.

## Background

Eukaryotic cell division is required for the separation of duplicated chromosomes and the subsequent step of physical division of the mother cells into two daughter cells. In plants, the process of chromosomal separation is generally similar to that of animal cells [1,2], whereas the mechanism of physical cell division, called cytokinesis, differs strikingly between plants and animals. In plants, the cell membrane-enveloped juvenile cell wall, referred to as the cell plate, accumulates between the daughter nuclei in late anaphase, and the cell plate subsequently expands centrifugally so as to divide the parental cell into two [3,4]. Cell plate formation and expansion are performed by structures that sandwich the developing cell plate, known as the phragmoplast, which is composed of membranes and cytoskeletons.

The cytokinetic membranes are generally thought to originate from Golgi-derived vesicles [3,5-7]. During cytokinesis, Golgi stacks were seen to accumulate and to become associated with the cell plate in living tobacco BY-2 cells [8] and in cryopreserved shoot apical meristematic cells of *Arabidopsis thaliana *[9]. It was recently proposed that endocytic vesicles also participate in cell plate formation [10], and this was based on the localization of several cell wall and/or plasma membrane (PM) materials in the cell plate; including xyloglucans [11,12], pectin [10,12], PM proteins, such as the auxin transport facilitators, PIN1 [13] and PIN2, the water channel, PIP2, the integral PM protein, LTI6b, and the brassinolide receptor, BRI1 [10], dynamin [10], clathrin [14], and the endosomal marker proteins, GNOM-Myc, Ara6 and Ara7 [10]. However, a more recent study demonstrated that endocytosis is not essential for cell plate formation [7]. The role of endocytosis in cell plate formation thus remains controversial.

Microtubules (MTs) are a major component of the phragmoplast, and the MTs and predicted kinesin motor proteins play a crucial role in the transport of cell plate materials [15,16]. The plus-ends of MTs face the cell plate [17] and terminate within the cell plate assembly matrix [6]. Recent electron tomographic analysis has revealed, with nano-scale resolution, that the three-dimensional geometry of phragmoplast MTs undergoes dynamic changes [18].

Actin microfilaments (MFs) are also distributed throughout the phragmoplast and are aligned parallel to the MTs, as reported in endosperm cells of the blood lily, *Haemanthus katherinae *[19-22], and the kaffir lily, *Clivia nobilis *[23], in root cells of onion, *Allium cepa *[24,25], in stamen hair cells of spiderwort plants, *Tradescantia virginia *[26-28], in cultured cells of carrot, *Daucus carota *[29], and alfalfa, *Medicago sativa *[30], as well as in tobacco BY-2 cells [31,32]. Electron microscopy-based, nano-scale structures of phragmoplast-related MFs have not yet been reported, mainly because of technical limitations [6]. Numerous studies have shown that, unlike MT inhibitors, actin inhibitors could not inhibit the initiation of the cell plate [27,33-37]. However, an actin polymerization inhibitor was found to delay cell plate expansion in *Haemanthus katherinae *endosperm cells [20]. Microinjection of profilin, a monomeric actin binding protein, into dividing *Tradescantia *stamen hair cells was found to disrupt phragmoplast-associated MFs and to inhibit cell plate expansion [27]. Furthermore, treatment with 2,3-butanedione monoxime (BDM), an inhibitor of myosin ATPase, or with ML-7, a specific inhibitor of myosin light-chain kinase, could delay the complete fusion of the cell plate with the parental cell wall [38]. Based on these results, MFs have been postulated to contribute to cell plate expansion at the late phase of cytokinesis. However, the mode of MF contribution to plant cytokinesis still remains unclear.

In this study, we have quantitatively estimated the time-sequential changes in MF contribution to cell plate development using live cell image analysis of tobacco BY-2 cell lines. In addition, we examined the relationship between MFs and endomembrane dynamics, including the endosomes and endoplasmic reticulum (ER). Our results detail new features of MFs in plant cytokinesis via regulation of the endomembrane system.

## Results

### Dynamic changes in MF localization during plant cytokinesis

To simultaneously observe MFs and the cell plate, tobacco BY-GF11 cells, in which MFs were visualized by expression of a GFP-ABD2 fusion protein [37], were stained with the styryl dye, FM4-64. After pulse-labeling with this endocytic tracer, the fluorescence moves from the PM to endosomes, and finally reaches the vacuolar membranes [39-41]. In addition, FM4-64 localizes to the cell plate membranes of *Arabidopsis *root cells [10,42] and tobacco BY-2 cells [40,43], in agreement with the previously suggested involvement of the endocytic pathway in membrane trafficking to the cell plate [10].

**Figure 1:**
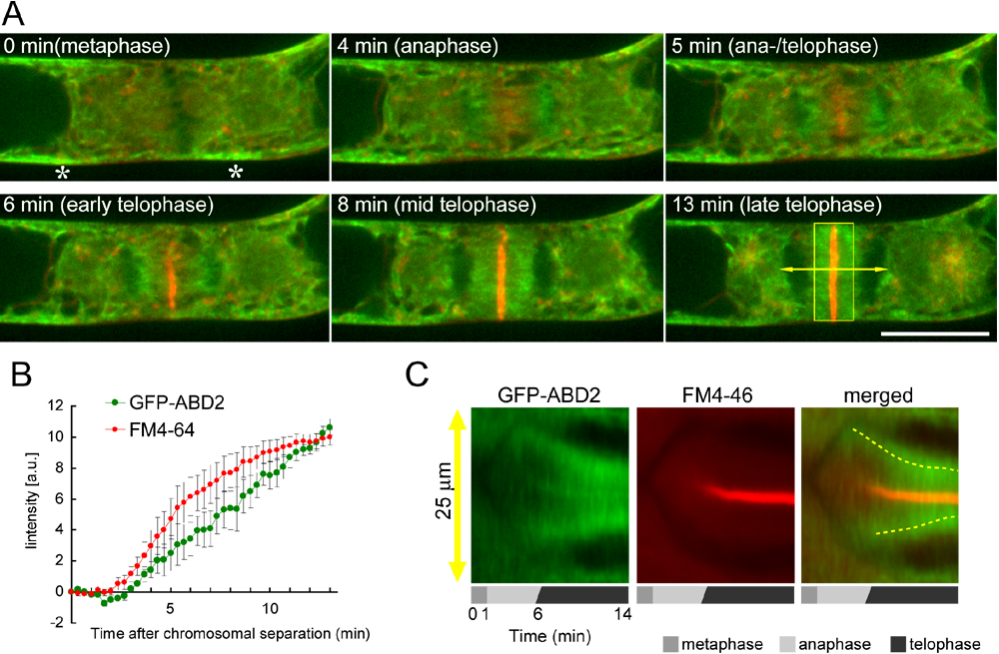
**Time-lapse imaging of actin microfilaments and cell plate**. **(A) **Time-lapse images of actin microfilaments and FM4-64-stained cell plates from metaphase to late telophase in a BY-GF11 cell. Green and red indicate GFP-ABD2 and FM4-64 fluorescence, respectively. Asterisks indicate actin microfilament twin peaks that appeared at metaphase [37]. We defined 0 min as the end of metaphase in this figure. Scale bar indicates 25 μm.**(B) **Changes in GFP-ABD2 and FM4-64 fluorescence intensities around the division plane. Intensities were time-sequentially measured along the division plane at a width of 9 μm, shown as the boxed region in (A). Data in (B) are mean values ± SE of four independent experiments. **(C) **Kymographs obtained by drawing a line across a cell plate from metaphase to late telophase, as shown by the yellow arrow in (A). Yellow broken lines indicate changes in MF localization during cell plate development. One representative experiment of four independent experiments is shown.

From 5 min to 3 h after pulse-labeling with 16 μM FM4-64, we observed that the fluorescence became localized mainly in the cell plate membranes and endosomes of the cytokinetic BY-GF11 cells (Fig. 1A), similar to that of wild type tobacco BY-2 cells (data not shown). At this time, there was still a slight remnant of fluorescence in the PM but not in the vacuolar membrane. For cell plate observations, all results presented in this report were, unless otherwise indicated, from those conducted 2 h after pulse-labeling.

At metaphase, no prominent MFs or FM4-64 fluorescence was observed in the mitotic apparatus (Fig. 1A, 0 min), whereas cortical MF twin peaks [37] were evident (Fig. 1A, 0 min, asterisks). From anaphase to telophase, the FM4-64 fluorescence gradually accumulated at the equatorial region, and the cell plate subsequently emerged (Fig. 1A, 4–6 min) as previously reported [10]. Concomitantly, there was a gradual accumulation of MFs from the periphery of the daughter nuclei towards the emerging cell plate (Fig. 1A, 4–6 min). At telophase, the MFs became localized near the expanding cell plate (Fig. 1A, 8–13 min). Measurements of fluorescent intensity around the division plane illustrate the accumulation of MFs in parallel with development of the FM4-64-labelled cell plate (Fig. 1B). To evaluate the spatial changes in MFs, a kymograph was constructed by tracking the fluorescence across the developing cell plate (Fig. 1C). The kymograph suggests that the MFs gradually approached the cell plate (Fig. 1C, yellow broken lines).

**Figure 2:**
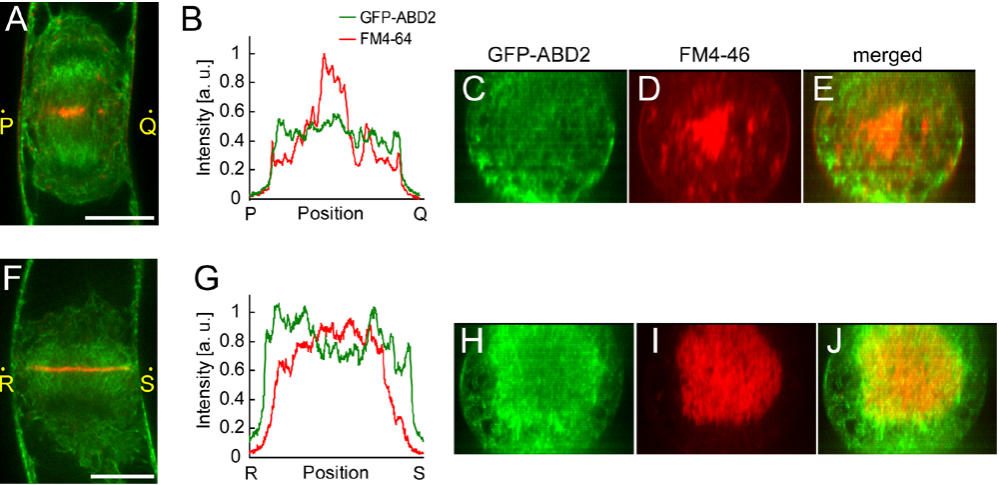
**Three-dimensional localization of actin microfilaments at early and late telophase**. **(A) **Single optical section of a BY-GF11 cell at early telophase. Green and red indicate GFP-ABD2 and FM4-64 fluorescence, respectively. **(B) **Intensity profiles of GFP-ABD2 and FM4-64 along the line segment PQ in (A).**(C) and (D) **Cross-sections of GFP-ABD2 (C) and FM4-64 (D) along the line segment PQ in (A). **(E) **Merged images of (C) and (D). **(F) **Single optical section of a BY-GF11 cell at late-telophase. Green and red indicate GFP-ABD2 and FM4-64 fluorescence, respectively. **(G) **Intensity profiles of GFP-ABD2 and FM4-64 along the line segment RS in (F). **(H) **and **(I) **Cross sections of GFP-ABD2 (H) and FM4-64 (I) along the line segment RS in (F). **(J) **Merged images of (H) and (I). Optical sections, which were used for reconstruction of a cross-section, were taken at 0.5 μm intervals. Scale bars indicate 10 μm.

To examine the structural relationship between MFs and cell plate during cytokinesis, the GFP-ABD2 and FM4-64 fluorescent intensities were measured along the cell plate at early (Fig. 2A, line segment PQ) and late (Fig. 2F, line segment RS) telophase. GFP-ABD2 was detected all along the equatorial plane, but its intensity was lower than that of FM4-64 in the cell plate at early telophase (Fig. 2B). In contrast, the intensity of GFP-ABD2 was high, especially at the edges of the cell plate, at late telophase (Fig. 2G). These results indicate that only a few MFs were localized in the mitotic apparatus at early telophase, but that they accumulated around the expanding cell plate at late telophase. A three-dimensional cross-sectional image, reconstructed from serial optical sections, confirmed these localization patterns at early (Fig. 2C, D and 2E) and late (Fig. 2H, I and 2J) telophase. These results suggest that MFs undergo dynamic changes in their arrangement during cell plate development.

### Quantitative analysis of the effects of an actin polymerization inhibitor on cell plate expansion

**Figure 3:**
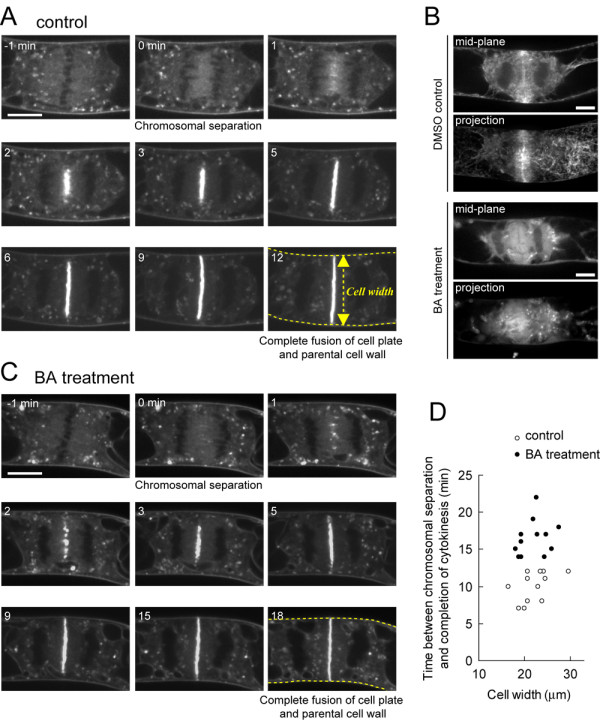
**Effects of bistheonellide A (BA) on the duration required to complete cytokinesis**. **(A) **Time-lapse images of FM4-64-labelled cell plate expansion in control cells. Time 0 min represents chromosomal separation. Yellow broken lines indicate parental cell wall. **(B) **Effect of BA treatment on MFs in cytokinetic BY-2 cells. BY-GF11 cells were treated with DMSO or 1 μM BA for 1 h. Representative images of a single optical section at the mid-plane (mid-plane) and maximum intensity projection (projection) are presented. **(C) **Time-lapse images of FM4-64-labelled cell plate expansion in BA-treated cells. Time 0 min represents chromosomal separation. Yellow broken lines indicate parental cell wall. **(D) **Effect of BA treatment on the duration between chromosomal separation and complete fusion of a cell plate and parental cell wall. Note that the duration times in BA-treated cells were longer than those in control cells, and were independent of cell size. The data were obtained from 12 independent experiments of each condition. Scale bars indicate 10 μm.

As mentioned above, vital staining with FM4-64 allowed us to follow chromosomal separation (Fig. 3A, -1-0 min), the gradual accumulation of cell plate vesicles (Fig. 3A, 1–2 min), cell plate expansion (Fig. 3A, 3–9 min), and final division of the parental cell (Fig. 3A, 12 min). To study the role of MFs in cell plate development, these sequential events were monitored under MF-disrupted conditions induced by pre-treatment with the dimeric macrolide, bistheonellide A (BA), an inhibitor of actin polymerization [44]. Treatment with 1 μM BA for 1 h almost completely destroyed the MF networks and resulted in the dispersion of free GFP-ABD2 fluorescence in the cytoplasm (Fig. 3B). A 1-h pre-treatment with 1 μM BA caused a temporary malformation of the emerging cell plate, which thus appeared to be discontinuous (as described further below; Fig. 3C, 2 min), and also increased the time needed for the expanding cell plate to complete its fusion with the parental cell wall independently of cell size (Fig. 3D). These results suggest that MFs partially contribute to cell plate formation and expansion.

To study the time-dependency of MF contribution to cell plate expansion, we sequentially measured the cell plate diameters from time-lapse images. Measurements of cell plate diameter and estimations of cell plate area showed that the expansion rate decreased with time in BA-treated cells (Table 1, BA treatment), whereas the rate was roughly constant in control cells (Table 1, control). These results suggest that the contribution of MFs to cell plate expansion increases with the progress of cytokinesis.

**Table 1:** Cell plate diameter and estimated cell plate area in tobacco BY-2 cells during cytokinesis.

**Time after initiation of cell plate formation (min)**	**Control**			**BA treatment**		
	
	**Diameter (μm)**	**Area (μm^2^)**	**ΔArea (μm^2 ^min^-1^)**	**Diameter (μm)**	**Area (μm^2^)**	**ΔArea (μm^2 ^min^-1^)**
0	0	0		0	0	
1	6.9 ± 0.3	37.9 ± 3.4	37.9 ± 3.6	ND	ND	ND
2	9.5 ± 0.5	72.2 ± 7.2	34.3 ± 6.2	ND	ND	ND
3	12.2 ± 0.5	119.2 ± 10.0	47.0 ± 6.6	11.2 ± 0.4	99.9 ± 8.2	ND
4	14.7 ± 0.6	172.1 ± 12.7	52.9 ± 5.2	13.7 ± 0.5	149.4 ± 11.9	49.5 ± 8.1
5	17.0 ± 0.6	229.3 ± 16.9	57.2 ± 5.6	15.3 ± 0.6	187.5 ± 16.2	38.2 ± 9.4
6	18.6 ± 0.6	274.0 ± 18.0	44.8 ± 6.1	16.5 ± 0.8	219.0 ± 20.0	31.5 ± 8.1
7	19.9 ± 0.6	313.5 ± 18.6	39.5 ± 4.6	17.7 ± 0.8	251.0 ± 23.0	31.9 ± 4.6
8	21.1 ± 0.7	352.5 ± 22.9	39.0 ± 7.8	18.4 ± 0.8	270.3 ± 23.4	19.3 ± 3.9
9	22.1 ± 0.9	389.2 ± 29.3	36.7 ± 7.4	18.9 ± 0.8	287.2 ± 24.7	16.9 ± 4.7
10	22.8 ± 1.0	427.3 ± 24.6	38.1 ± 7.7	19.6 ± 0.8	306.5 ± 27.1	19.3 ± 5.0
11				20.0 ± 0.9	321.0 ± 28.6	14.5 ± 3.0
12				20.3 ± 0.9	330.1 ± 29.0	9.1 ± 1.9
13				20.7 ± 0.9	342.0 ± 33.6	11.9 ± 3.0
14				21.1 ± 1.0	359.1 ± 32.1	17.1 ± 6.0
15				21.7 ± 0.9	376.4 ± 32.5	17.3 ± 7.5
16				21.9 ± 0.9	383.4 ± 34.3	7.0 ± 3.4

ND: Not defined.

**Figure 4:**
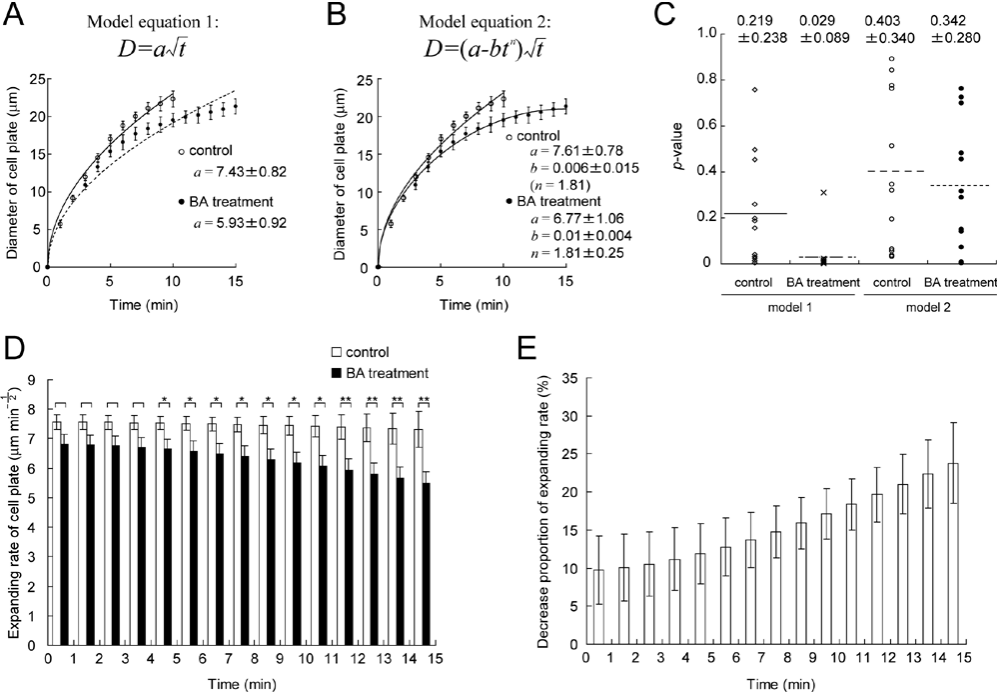
**Quantitative analysis of effects of bistheonellide A (BA) on cell plate expansion**. **(A) and (B) **Regression analysis of the data for changes in cell plate diameter of control (open circle) and BA-treated (filled circle) cells. Time 0 min represents the initiation of cell plate formation. Values are arithmetic means ± SE from 12 independent experiments. The continuous and broken lines represent the regression curves for cell plate diameter of control and BA-treated cells, respectively, calculated from model equation 1 (A) or 2 (B). In model equation 1, the expansion rate *a *is constant (A). In model equation 2, the expanding rate *a-bt*^*n *^undergoes time-dependent changes (B). When the control data was applied to model equation 2, *n *became underspecified and was consequently assumed to be invariant at 1.81. **(C) **Comparison of *p*-values in chi-square goodness of fit tests. The *p*-value indicates the probability that an observed difference between the measured and predicted values by model equations 1 or 2 occurred by chance alone. If the *p*-value is less than the significant level at 0.05, we cannot accept that there are no differences between the measured and predicted values. Note that the data fit model equation 2 (B) much better than model equation 1 (A). **(D) **Simulation of changes in the effects of BA treatment on expansion rate calculated at 1 min intervals. The values are arithmetic means ± SE from 12 independent calculation results of *a-bt*^*n*^. The 12 parameter sets *a *and *b *were the same as calculated in (B). Significance was determined using Student's t-test. *p*-value *< 0.05. **<0.001. **(E) **Simulation of changes in the estimated contribution of MFs to cell plate expansion. Values are arithmetic means ± SE from 12 independent calculated results of the decrease proportions of the expanding rate. The decreased proportion was calculated as the ratio of the difference between *a-bt*^*n *^in a control and a BA-treated cell to the *a-bt*^*n *^in a control cell. The values of *a-bt*^*n *^were the same as calculated in (D). Pairing of the control and BA-treated data was performed under the condition that the difference of *a *in a control and a BA-treated cell was at a minimum value.

**Figure 5:**
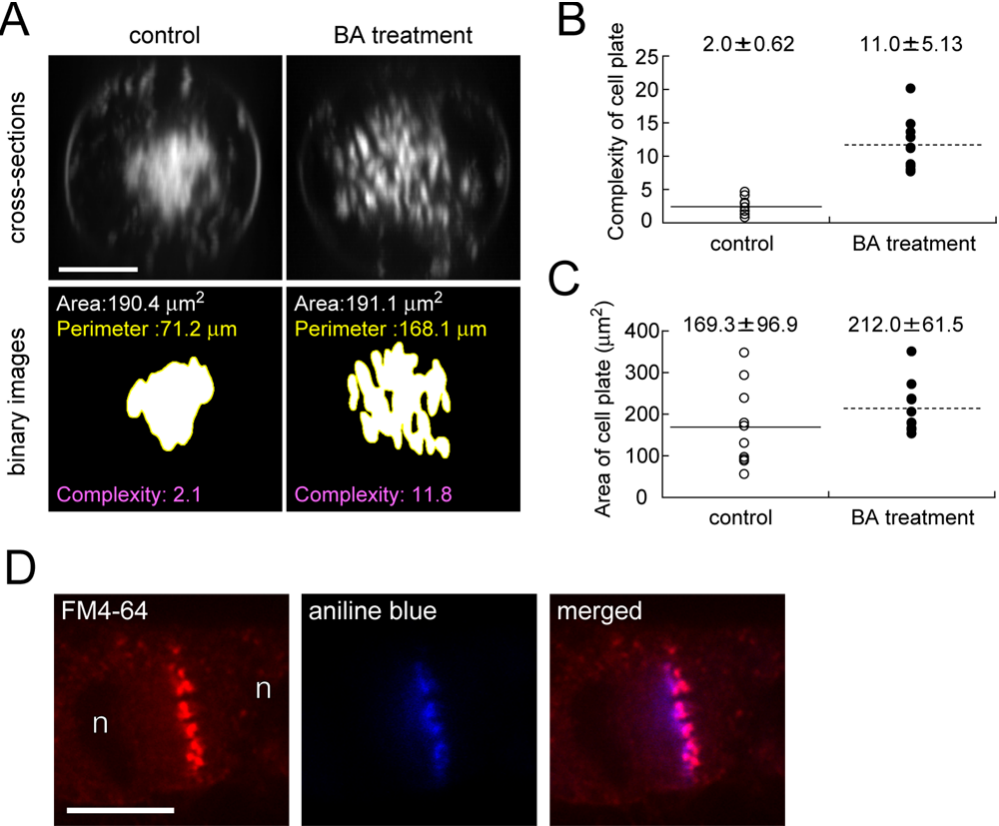
**Effects of bistheonellide A (BA) on morphology of emerging cell plates**. **(A) **Morphometry of cell plates. To define the cell plate configuration from a cross-section (upper images) of an FM4-64-labelled cell plate, a binary image (lower images) was obtained by intensity thresholding. From the binary image, the cell plate area and perimeter were measured. Complexity was calculated from the area and perimeter (see Methods for details). **(B) and (C) **Cell plate complexity (B) and area (C). Results from 10 cells in each condition are shown.**(D) **Double-staining with FM4-64 and aniline blue in fixed cells. n indicates daughter nuclei. Scale bars indicate 10 μm.

The diameter of the FM4-64-stained cell plate was measured as shown in Figure 4A. Values are arithmetic means ± SE, n = 12. Cell plate area was estimated by assuming that the cell plate is a perfect circle (*Area *= *π *(*Diameter*)^2^/4). Diameters at 1 and 2 min after initiation of cell plate formation (Table 1, BA treatment) could not be defined because the emerging cell plate was deformed in BA-treated cells (see Fig. 3C, 2 min and Fig. 5).

To quantitatively estimate this contribution of MFs, we used a simple mathematical model for cell plate expansion, based on the well-known concept of a constant increase in cell plate area [45]. In turn, the diameter increased with the square root of time, as follows:

(1) $$D=a\sqrt{t}$$

where *D *is the cell plate diameter, *a *is the expansion rate, and *t *is time. The time-lapse data of the diameter fit equation 1 well for the control cells (Fig. 4A, control) but not for the BA-treated cells (Fig. 4A, BA treatment), as demonstrated by the chi-square goodness of fit test (Fig. 4C, model 1). This result suggests that disruption of MFs affected the constancy of cell plate expansion.

Consequently, we developed a new model equation as given by:

(2) $$D=(a-bt^{n})\sqrt{t}$$

where *a*, *b *and *n *are constants. In this model, the expansion rate, *a-bt*^*n*^, gradually slows if both *b *and *n *are positive. In BA-treated cells, the time-lapse data precisely fit this model 2 (Fig. 4B, BA treatment, Fig. 4C). The mean values of *a*, *b *and *n *were 6.77, 0.01 and 1.81, respectively (Fig. 4B, BA treatment). These results indicate that the expansion rate was reduced in BA-treated cells and suggest that MFs make a gradual contribution to cell plate expansion.

However, to compare the contributions of MFs to cell plate expansion in control and BA-treated cells, both data sets need to be applied to the same model equation. When we applied time-lapse data from control cells to model equation 2, *n *became underspecified because *b *came close to 0 (data not shown). We, therefore, assumed that *n *is invariant at 1.81, even in the control cells, and applied the control data to model equation 2 (Fig. 4B, control). Consequently, the control data precisely fit model 2 as well as model 1 (Fig. 4C). The mean values of *a *and *b *were 7.61 and 0.006, respectively (Fig. 4B, control). Simulating the expansion rate of the cell plate with model equation 2 quantitatively estimated the changes in the expansion rate (i.e. *a-bt*^*n *^in model equation 2) during cytokinesis in control and BA-treated cells (Fig. 4D). Finally, the contribution of MFs to cell plate expansion could be time-sequentially estimated by the differences between expansion rates in control and BA-treated cells (Fig. 4E). The contribution of MFs to cell plate expansion was found to be about 10% just after cell plate formation and gradually increased up to about 25% at the end of cytokinesis (Fig. 4E).

### MF-disruption-induced a temporary malformation of the emerging cell plate

Interestingly, a 1 h pre-treatment with 1 μM BA caused malformation of the emerging cell plate, which thus appeared discontinuous (Fig. 3C, 2 min). This aberration was only temporary, in that it could be observed for a maximum of only 1–2 min (Fig. 3C, 2 min), after which the blank areas of the cell plate were filled in so that it attained a normal appearance (Fig. 3C, 3–5 min). Cross-sectional images revealed abnormal structures with several holes (Fig. 5A, upper panels). To assess their morphology, we extracted the cell plate area from the cross-sections (Fig. 5A, lower panels), and measured their area, perimeter and complexity using ImageJ software [46]. Complexity was defined as a morphological parameter and calculable from the area and perimeter (see Methods) [47]. A circle has the lowest value, 1.0, of complexity and, as the structure becomes more complicated, the complexity reaches appropriately higher values [47]. The mean value of complexity of cell plates in BA-treated cells was higher than that of control cells (Fig. 5B), whereas their mean areas did not differ significantly (Fig. 5C). In addition, aniline blue staining revealed that the deformed cell plate contained callose (Fig. 5D) as in the control cells (data not shown). These results suggest that MFs modulated cell plate formation/expansion at the early phase of cytokinesis, but did not affect callose synthesis.

### Actin-myosin-dependent endosomal movement around the cell plate

**Figure 6:**
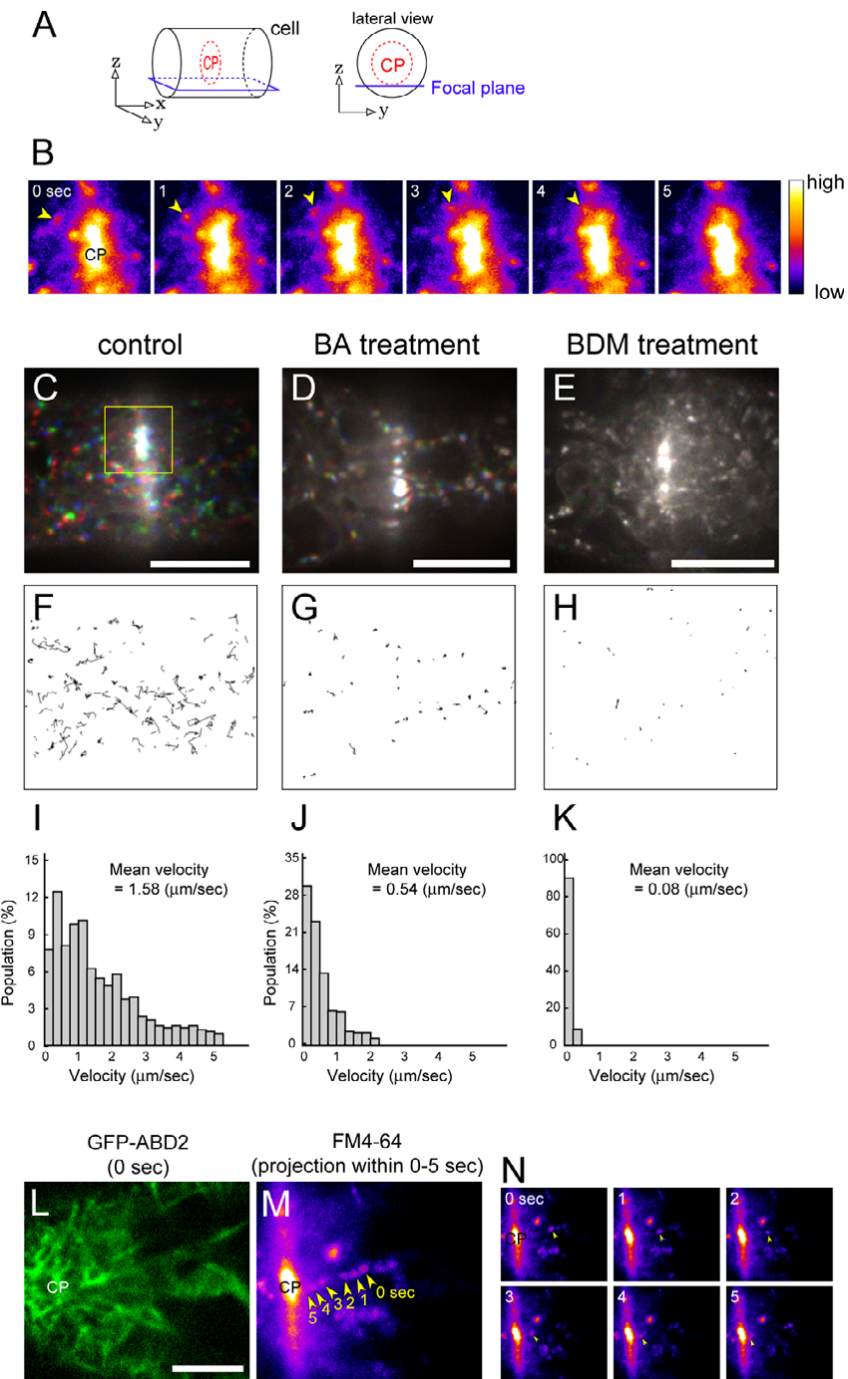
**Effects of bistheonellide A (BA) and 2,3-butanedione monoxime (BDM) on endosome movement around the expanding cell plate edge**. **(A) **Schematic representation of the focal plane for image capture around the edge of an expanding cell plate. All microscopic images presented in this figure were focused at the tangential surface of the cell plate. CP represents the cell plate. **(B) **Interaction of endosomes with the edge of a cell plate. Magnified and time-sequential images of the boxed region in (C) are shown. To facilitate endosome visualization, images are presented in pseudo-color. Movement of an endosome (yellow arrowheads) towards the cell plate (0–4 sec) and merger with the edge (5 sec). **(C)-(E) **Movement of endosomes stained with FM4-64 around a cell plate in control (C), BA-treated (D) and BDM-treated (E) cells. Images at 0, 15 and 30 sec are colored in red, green, and blue, respectively, and projected together. Scale bars indicate 10 μm.**(F)-(H) **Tracking of endosomal movement in control (F), BA-treated (G) and BDM-treated (H) cells. Confocal sections were taken at 1-sec intervals for 30 sec, and the endosomes were tracked by ImageJ software (see Methods). **(I)-(K) **Frequency histograms of endosome velocity over a 30 sec period. Data were obtained from 599 (I), 488 (J), 382 (K) endosomes from 10 control (I), 18 BA-treated (J), and 15 BDM-treated (K) cells, respectively. **(L)-(N) **Dual observations of MFs (L) and moving endosomes (M and N). To facilitate endosome visualization, images are presented in pseudo-color as in (B). MF structures around the cell plate edge (L) and endosome movement (N, yellow arrowheads) were observed simultaneously. To facilitate visualization of the movement, maximum intensity projections of the time-sequential images are presented (M). Note the movement of an endosome towards the cell plate along the MFs. CP represents the cell plate. Scale bar indicates 5 μm.

To investigate the interactive mechanisms of MFs and cell plate formation/expansion, the relationship between MFs and endosomes, which have been implicated in cell plate development [10], was examined. In a control cell at the late phase, there was active movement of numerous endosomes (Fig. 6C and 6F) around the edge of the expanding cell plate (Fig. 6A). Some endosomes were found to interact with the edge of the expanding cell plate (Fig. 6B). To examine the role of actin-myosin systems on endosomal movement around the cell plate, we treated the cells with BA or 2,3-butanedion monoxime (BDM), a general myosin ATPase inhibitor [48,49]. The movements were discernibly inhibited by BA (Fig. 6D and 6G) and BDM pre-treatments (Fig. 6E and 6H). These inhibitory effects were confirmed statistically by measuring the velocity of the endosomal movements (Fig. 6I, J and 6K). Dual observations of MFs and endosomes showed that the endosomes moved towards the cell plate along the MFs (Fig. 6L, M and 6N). These results suggest that endosomal movement around the edge of the expanding cell plate depends on the actin-myosin systems.

### MF-dependent accumulation of ER in the phragmoplast

**Figure 7:**
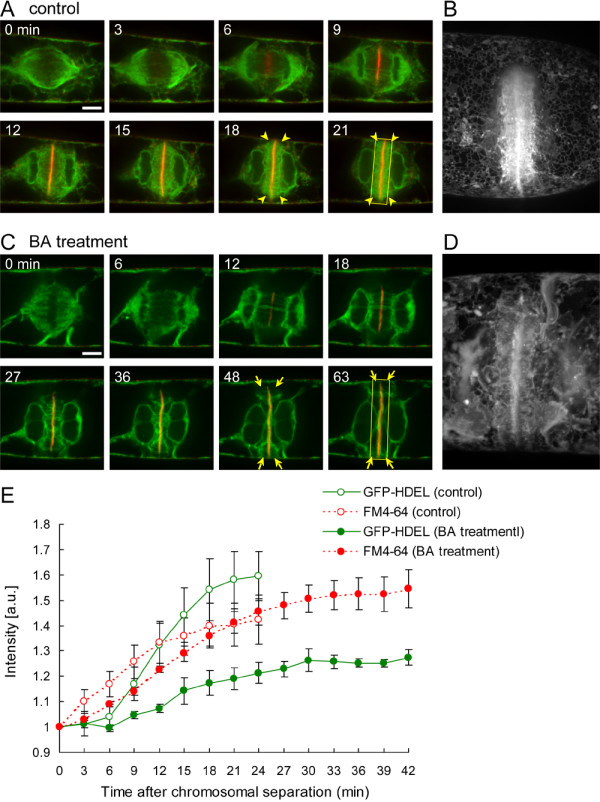
**Effects of bistheonellide A (BA) on organization of phragmoplast-related endoplasmic reticulum (ER)**. **(A) **Time-lapse images of GFP-labeled endoplasmic reticulum (ER) and FM4-64-labeled cell plate during cytokinesis in a control cell. ER accumulated around the edge of the expanding cell plate before completion of cytokinesis (yellow arrowheads). **(B) **Projection image of ER at late telophase in a control cell. **(C) **Time-lapse images of GFP-labeled ER and FM4-64-labeled cell plate in a BA-pretreated cell. Accumulation of ER in the phragmoplast was inhibited (yellow arrows) compared with the control cell shown in (A). **(D) **Projection image of ER at late telophase in a BA-treated cell. **(E) **Changes in GFP and FM4-64 fluorescence intensities around the division plane. Intensities were time-sequentially measured in the division plane at a width of 9 μm, as shown in the boxed region of (A) and (C). Data in (E) are mean values ± SEs of four independent experiments.

To further test the involvement of MFs in endomembrane dynamics during cytokinesis, we analyzed the effects of BA treatment on ER-organization during cytokinesis in tobacco BY-2 cells expressing SP-GFP-HDEL, which is retained within the ER [50]. ER accumulated in the spindle poles in meta-/anaphase (Fig. 7A, 0–3 min), sandwiched the cell plate in telophase (Fig. 7A, 6–21 min), and accumulated in the phragmoplast in late telophase (Fig. 7A, 18–21 min, yellow arrowheads), as previously reported [6,8,51]. A 1 h BA pre-treatment did not induce marked changes in ER-organization at metaphase (Fig. 7C, 0 min), but did inhibit ER-accumulation in the phragmoplast at late telophase (Fig. 7C, 48–63 min, yellow arrows). Three-dimensional images also showed a significant decrease in the amount of ER in the phragmoplast (Fig. 7B and 7D). In addition, time-sequential measurements of fluorescent intensity around the division plane illustrated the inhibition of ER-accumulation in the phragmoplast (Fig. 7E).

## Discussion

In this study, we have quantitatively examined the temporary changes in MF contribution to cell plate expansion. To visualize the MFs and cell plate, we used GFP-ABD2 expression and FM4-64 vital staining, respectively. Although GFP-ABD2 is recognized as a more reliable MF-marker than GFP-mouse talin (GFP-mTn) [37,52-54], recent studies have shown that overexpression of GFP-ABD2 can cause growth inhibition of pollen tubes [55] and organelle motility [56], suggesting the need for care and attention in the use of such GFP markers. Our BY-GF11 cell line shows a normal growth rate compared with wild-type BY-2 cells (data not shown) and appears to have minimal side effects. Furthermore, FM4-64, which has been widely used as an endocytosis marker, was found to induce abnormal membrane systems in fungal cells [57]. However, FM4-64 cytotoxicity appears limited in plant cells [40] and, at least under our experimental conditions [39], abnormal membrane structures or an inhibition of cell division in the BY-2 cells were not observed. These labeling techniques, which cause minimal cell damage, have allowed us to study MF-dynamics and their roles in cell plate development.

### MFs contribute to cell plate expansion in late expansion phase, but even in early initiation phase

It is widely accepted that there are two stages in plant cytokinesis that can be distinguished by cell morphological features and sensitivity to caffeine [28]. The first stage is the early initiation phase, which starts immediately after chromosomal separation and involves the deposition and early expansion of the cell plate until it reaches the width of the daughter nuclei. The second stage is the late expansion phase that involves growth of the cell plate beyond the width of the nuclei until it reaches and fuses with the parental cell wall. The late expansion phase is completely inhibited by caffeine [28], which also inhibits callose deposition and the organization of phragmoplast MTs [58].

MFs are thought to be also involved in the late expansion phase of the cell plate. In cytochachalasin-treated *Haemanthus katherinae *endosperm cells, the cell plate often remains incomplete [20]. Similar cell plate inhibition has been reported in profilin-microinjected *Tradescantia virginiana *stamen hair cells [28], and in *Tradescantia *stamen hair cells treated with myosin inhibitors [38]. In this study, the increase in MF contribution to the late expansion phase of the cell plate, which was confirmed by time-sequential measurements and data fitting analysis (Fig. 4), concurs with previous reports and also with the gradual accumulation of MFs near the cell plate found in this study (Fig. 1C). On the other hand, our results revealed that MF-disruption decreased the expansion rate by about 10%, even just after cell plate formation, but by about 25% at the end of cytokinesis (Fig. 4E) and, in addition, caused malformation of the emerging cell plate (Fig. 5). These results suggest that MFs not only facilitate cell plate expansion in the late expansion phase but also in the early initiation phase.

Furthermore, MF-disruption-induced malformation of the cell plate suggests that MFs regulate the early disc-like morphology of the cell plate. In the early phase, the MFs had not yet accumulated near the cell plate, but a thin, widely-spread MF-meshwork could be observed in the mitotic apparatus and near the cell plate (Fig. 1A, Fig. 2A–E). The existence of MFs near the cell plate in the early phase was also observed by rhodamine-phalloidin staining of fixed cells of onion *Allium cepa *[24]. BA-induced cell plate malformation is reminiscent of the mini-phragmoplasts, which appear during *Arabidopsis *endosperm cellularization [59,60] and in post-meiotic cytokinesis during pollen development [61]. Mini-phragmoplasts consist of 2 to12 overlapping MT clusters, assembled between duplicated nuclei, and spaced on average 0.6 μm apart from each other across a given division plane [61]. BA-induced cell plate blank regions were approximately of the same size (Fig. 5A, BA treatment). The fact that similar structures occur by MF-disruption suggests that MFs are involved in the spread and flattening of the cell plate at the early phase.

### Actin-myosin system is possibly involved in plant cytokinesis via endosomal dynamics

In this study, we found MF-disruption had parallel, aberrant effects on cell plate development and endosomal movement around the expanding cell plate (Fig. 6D, G and 6J). Treatment with the general myosin inhibitor, BDM, revealed that endosomal movements depended on the actin-myosin system (Fig. 6E, H and 6K). MF-dependent endosomal dynamics were also reported in root cells of *Arabidopsis thaliana *[13,62-64], *Zea mays *[65,66], *Medicago truncatula *[67] and pollen tubes of *Picea meyeri *[68]. Endosome association with MFs was observed in cytokinetic BY-2 cells (Fig. 6L, M and 6N) as well as in root hair cells [67]. From these results, we hypothesize that the actin-myosin systems are partially involved in endocytic membrane traffic to the cell plate. This notion is supported by recent studies demonstrating that the plant myosin class VIII, ATM1, is localized in the expanding cell plate of tobacco BY-2 cells [69] and in FM4-64-labelled endosomes of *Nicotiana benthamiana *leaves [70]. Although the endocytic pathway might participate in membrane trafficking to the cell plate [10], it is not essential for completion of cytokinesis [7]. Indeed, as Golgi-derived vesicles transported by MTs and unidentified kinesins are necessary and sufficient for cell plate development [7,15], the contribution of MFs to cell plate formation and expansion may be rather limited. Unfortunately, we could not examine the effects of BDM on cell plate expansion, because BDM-pretreatment inhibited cell division in the BY-2 cells (data not shown), possibly through their side effects on MTs [48]. Future studies, using combinations of genetic inhibition of myosin activity and time-sequential morphometry as shown in this study, will reveal the changes in contribution of myosins to cell plate expansion.

### MFs regulate ER-accumulation in the phragmoplast at late expansion phase

The ER is one of the first organelles to be visualized during plant cytokinesis. Numerous electron microscopic studies have shown that the tubular ER is first arranged parallel to the spindle MTs in metaphase, and then gradually accumulates near the cell plate and phragmoplast region [6,71,72]. Recent studies on the expression of ER-targeted fluorescence proteins have further described the dynamic behavior of the ER in living cells [8,51]. Generally, the complex cortical ER-network structures are found to depend on MFs in plant cells [73,74] but on MTs in animal cells [75,76]. On the other hand, ER-organization in mitotic spindles is associated mainly with MTs rather than MFs in mitotic cells of the gymnosperms, *Pinus brutia *and *Pinus nigra *[77], and in tobacco NT-1 cells [51]. Our observation that the tubular ER aligned with mitotic spindles in BA-treated cells (Fig. 7C, 0 min) supports these earlier reports. In addition, we found that BA treatment inhibited ER-accumulation in the phragmoplast, especially in the late expansion phase (Fig. 7C, 48–63 min, D and E), suggesting that MFs are tightly associated with ER recruitment in the phragmoplast. In dividing tobacco mesophyll protoplasts, an unbiased ER redistribution was also found to be dependent on MFs [78].

The ER has been implicated in the control of the cytosolic calcium ion concentrations [79] and in facilitation of membrane exchange between the ER and cell plate during cell plate maturation [6]. Therefore, it is likely that MFs contribute to cell plate expansion via recruitment of ER into the phragmoplast, and this is supported by the observed increase in MF contribution at the late phase. Taken together with the results from endosomal dynamics, these findings thus imply that MFs have multiple functions in the endomembrane systems during cytokinesis. Future studies will need to focus on molecular dissection of the complicated interactions between MFs and endomembranes to gain further insights into MF contribution to plant cytokinesis.

## Methods

### Plant material and cell synchronization

Tobacco BY-2 (*Nicotiana tabacum *L. cv. Bright Yellow 2) cells and BY-GF11 cells expressing GFP-ABD2 [37] were diluted 95-fold with a modified Linsmaier and Skoog medium at weekly intervals [80]. The cell suspensions were agitated on a rotary shaker at 130 rpm at 27°C in the dark. For cell synchronization, 10 ml of 7-day-old cells were transferred to 95 ml fresh medium and cultured for 24 h with 5 mg liter^-1 ^aphidicolin (Sigma Chemical Co., St. Louis, MO, USA) [81]. The cells were washed with 10 volumes of fresh medium and resuspended in the same medium.

### Microscopy

The cells were transferred into φ35 mm Petri dishes with φ14 mm coverslip windows at the bottom (Matsunami, Osaka, Japan). The dishes were placed onto the inverted platform of a fluorescence microscope (IX-70; Olympus) equipped with a CSU10 scanning head (Yokogawa, Tokyo, Japan) and a cooled CCD camera head system (CoolSNAP HQ, PhotoMetrics). For multifocal observations, serial optical sections were taken at 0.5-μm intervals.

### Cell staining

To observe the cell plate and endosomes, *N*-(3-triethylammoniumpropyl)-4-(6-(4-(diethylamino) phenyl) hexatrienyl) pyridinium dibromide (FM4-64; Molecular Probes, Invitrogen) was added to the cell suspension at a final concentration of 16 μM. The cells were incubated for 2 min, washed with fresh culture medium, and then monitored. To simultaneously stain the cell plate and callose, the FM4-64-pre-stained cells were fixed with 3.7% (w/v) formaldehyde dissolved in PIPES buffer (pH 6.8) containing 1 mM MgSO_4_, 5 mM EGTA and 1% glycerol (PMEG) for 1 h, and then stained with 0.05% aniline blue (Biosupplies Australia, Parkville, Victoria, Australia) in PMEG for 30 min.

### Inhibitor

For disruption of MFs, cells were treated with 1 μM bistheonellide A (BA, Wako Pure Chemical Ind., Osaka, Japan) for 60 min before observations. Myosin activity was inhibited by treatment with 20 mM BDM (Sigma Chemical Co., St. Louis, MO, USA).

### General Image analysis

Indirect quantification of fluorescence levels, maximum intensity projections and kymograph construction were performed using ImageJ software [46]. Kymographs were constructed with our in-house developed plug-in package for ImageJ software, which is freely available from our website .

### Time-sequential measurements of cell plate diameter

To measure the diameter of an expanding cell plate, a binary image was obtained by intensity thresholding and used for measurements on ImageJ. A Gaussian filter was applied to the cross-sectional images before binarization. Cell plate diameters were calculated with ImageJ software. Regression analysis of the cell plate diameter was performed using Kaleida-graph software (Synergy Software, Reading, PA).

### Assessment of cell plate morphology

To assess the cell plate structures, we first obtained cross-sectional images from 70 serial optical sections taken at 0.5 μm intervals of each cell with view orthogonal planes function in MetaMorph image-analysis software (Universal Imaging Co., Downingtown, Panama). Subsequently, we obtained the binary images from the cross-sectional images by intensity thresholding using ImageJ software as shown in Figure 5A. To improve the signal to noise ratio, a Gaussian filter was applied to the cross-sectional images before binarization. Cell plate area and perimeter were calculated from the binary images with ImageJ software. Complexity, a secondary morphological parameter, was calculated by P^2^/4πA, where P is the perimeter and A is the area, according to Tanaka et al. (2007) [47].

### Analysis of endosomal movement

To estimate the velocity of endosomal movement, we performed time-sequential observations at 1-sec intervals for 30 sec by spinning-disc confocal laser microscopy. From the time-sequential images, we measured the distance and time traveled by endosomes using the ImageJ plug-in MTrack2, which was developed for multiple particle detection and tracking, and which is freely available from the website . Subsequently, endosomal velocity was calculated by dividing the distance traveled by the traveling time.

## Abbreviations

ABD2: actin binding domain 2; BA: bistheonellide A; BDM: 2,3-butanedion monooxime; ER: endoplasmic reticulum; GFP: green fluorescent protein; MF: actin microfilament; MT: microtubule; PM: plasma membrane.

## Authors' contributions

TH conducted all the experiments and wrote drafts of the manuscript. NK helped in image analyze and preliminary experiments of cell plate membrane dynamics. SH oversaw the project in his laboratory and is the guarantor of the work. All authors read and approved the final manuscript.
